# Potential bioactivities via anticancer, antioxidant, and immunomodulatory properties of cultured mycelial enriched β-D-glucan polysaccharides from a novel fungus *Ophiocordyceps sinensis* OS8

**DOI:** 10.3389/fimmu.2023.1150287

**Published:** 2023-04-11

**Authors:** Suwannachom Chatnarin, Mongkol Thirabunyanon

**Affiliations:** Program in Biotechnology, Faculty of Science, Maejo University, Chiang Mai, Thailand

**Keywords:** anticancer, antioxidant, colon cancer, immunomodulatory, *Ophiocordyceps sinensis*, polysaccharides

## Abstract

**Background:**

*Ophiocordyceps sinensis* is well-known worldwide as a traditional medicine. An alternative natural source of *O. sinensis* is provided by mycelial cultivation. However, the bioactivities of cultured mycelial-enriched β-D-glucan polysaccharides from a novel fungus *O. sinensis* OS8 are still unknown.

**Methods:**

We investigated the potential bioactivities via anticancer, antioxidant, and immunomodulatory polysaccharides (OS8P) produced from cultured mycelia of *O. sinensis* OS8. This strain is a novel fungus isolated from natural *O. sinensis*, which is further cultured by submerged mycelial cultivation for polysaccharide production.

**Results:**

The yield of mycelial biomass was 23.61 g/l, and it contained 306.1 mg/100 g of adenosine and 3.22 g/100 g of polysaccharides. This OS8P was enriched with β-D-glucan at 56.92% and another form of α-D-glucan at 35.32%. The main components of OS8P were dodecamethyl pentasiloxane, 2,6-bis (methylthiomethyl) pyridine, 2-(4-pyrimidinyl)-1H-Benzimidazole, and 2-Chloro-4-(4-nitroanilino)-6-(O-toluidino)-1,3,5-triazine at the rates of 32.5, 20.0, 17.5, and 16.25%, respectively. The growth of colon cancer cells (HT-29) was significantly inhibited by OS8P, with IC_50_ value of 202.98 µg/ml, and encouraged apoptosis in HT-29 cells as confirmed by morphological change analysis via AO/PI and DAPI staining, DNA fragmentation, and scanning electron microscopic observations. In addition, significant antioxidant activity was demonstrated by OS8P through DPPH and ABTS assays, with IC_50_ values of 0.52 and 2.07 mg/ml, respectively. The OS8P also exhibited suitable immunomodulatory activities that significantly enhanced (*P<* 0.05) the induction of splenocyte proliferation.

**Conclusion:**

The OS8P enriched with β-D-glucan polysaccharides and produced by submerged mycelial culture of a new fungal strain of *O. sinensis* OS8 strongly inhibited the proliferation of colon cancer cells without any cytotoxicity against normal cells. The potential effect of the OS8P on the cancer cells was due to the stimulation of apoptosis. Also, the OS8P exhibited good antioxidant and immunomodulatory activities. The results indicate that OS8P has promising applications in the functional food industry and/or therapeutic agents for colon cancer.

## Introduction

In recent years, there has been a surge of interest in studying natural compounds with potential chemopreventive and chemotherapeutic effects against human diseases. Colon cancer is the third most common cancer (10.0%) and the second most fatal (9.4%) malignant cancer (1). A multitude of studies has highlighted the presence of bioactive molecules in various natural sources, such as fruits, vegetables, herbs, and spices, that possess potent anti-inflammatory, antioxidant, and anticancer properties. These compounds have demonstrated the ability to regulate multiple cellular signaling pathways involved in the expansion and progression of colon cancer. As a result, they hold immense promise as a means of inhibiting tumor growth, inducing cancer cell death, and reducing the likelihood of cancer recurrence. Exploring the use of natural compounds as possible agents for colon cancer restraint and therapy presents an exciting avenue for research, offering a safer and potentially more effective alternative to conventional chemotherapy and radiotherapy. Furthermore, several dysfunctions in the human body with many adverse long-term health effects, such as bladder and bowel dysfunctions, coincide with current therapies (2). Thus, developing highly efficient bioprophylactic and biotherapeutic agents with low side effects is urgently needed.

*Ophiocordyceps sinensis* (former: *Cordyceps sinensis*) is a type of entomopathogenic fungi whose fruiting bodies or mycelia have been extensively studied for their diverse biological activities (3). This fungus, also referred to as summer grass or winter worm, is typically found in prairie soil at high altitudes ranging from 3,500 to 5,000 meters (4). Due to its many bioactive compounds, such as carbohydrates, proteins, adenosine, and cordycepin, this fungus is a traditional medicine used in many countries to prevent and treat various diseases (5). Since it comprises many bioactive substances including carbohydrates, protein, adenosine, and cordycepin (6), it continues to attract significant attention from researchers and has become an important subject of investigation in the field of natural product research.

Polysaccharides are long-chain polymers comprised of aldehyde and ketone groups that are linked together via glycosidic bonds. These compounds are among the original bioactive molecules found in medicinal mushrooms and are known to exhibit a diverse array of biological activities. In particular, polysaccharides derived from *C. sinensis* have demonstrated significant antitumor, antioxidant, and immunomodulatory effects (7). Similarly, the extracted mycelial polysaccharides from *O. sinensis* have been found to possess strong antioxidant properties and offer health benefits in protecting against oxidative damage (8). Additionally, β-D-glucan, an exopolysaccharide from cultured mycelia of *C. sinensis*, exhibits strong antioxidant activity (9). Polysaccharides derived from *C. sinensis* have been proposed to possess neuroprotective properties, which may aid in preventing free radical-mediated harm to neuronal cells (10). Given their numerous potential health benefits, polysaccharides from these fungi continue to be the focus of intense research and investigation in the pursuit of natural products with medicinal chemistry.

Immunomodulators are substances that can improve the efficacy of the body’s immune system, enabling it to better control and eliminate cancer cells through the adaptive immune response conducted by lymphocytes, a type of white blood cell (11, 12). This type of immune response involves two broad classes of antibody and cell-mediated immune responses, which are carried out by B cells and T cells, respectively (13). Immunomodulatory compounds can assist to enhance the functions of these lymphocytes and enhance the overall immune response against cancer. In addition to their immunomodulatory effects, polysaccharides from *C. sinensis* have been found to contain intracellular selenium obtained by mycelia of *C. sinensis* SU-02 in submerged culture. This compound has potent antioxidant properties and has been shown to enhance the adaptive immune response (14). These findings offer promising avenues for further research into the development of natural compounds as potential immunomodulators.

Polysaccharide compounds found in different mushroom species exhibit diverse bioactivities, which can vary even within the same species. These differences can be attributed to various factors such as the origin of the mushroom, environmental habitat, and the media in which the mushroom is cultured. In light of these factors, a novel strain of *O. sinensis* OS8 was isolated from natural sources of *O. sinensis* and utilized in liquid fermentation to produce mycelial biomass. In this study, polysaccharides obtained from the submerged mycelial culture of *O. sinensis* OS8 were examined for their structural configuration, bioactive compound composition, their prospective to proliferative inhibition of colon cancer cells, cell apoptosis, and antioxidant as well as immunomodulatory activities. This research offers a promising approach for investigating the therapeutic potential of novel strains of *O. sinensis*, as well as for identifying and characterizing the bioactive compounds responsible for the observed effects. By examining the structural and functional properties of polysaccharides from medicinal mushrooms, researchers can gain a deeper understanding of their potential health benefits and develop more effective natural therapies for cancer and other diseases.

## Materials and methods

### Mycelial gathering and isolation

The 119 dried samples of *O. sinensis* natural fruiting bodies were collected from various regions in China, including Yunnan and Gansu (20 samples), Qinghai (20 samples), Sichuan (39 samples), and Tibet (40 samples). Each sample of *O. sinensis* fruiting body was carefully washed with sterile water and then soaked in a 1% sodium hypochlorite solution (Merck, Germany) for one min. Next, the sample was cut into small pieces, measuring 2 - 5 mm in length, and then inoculated onto SDA agar. The inoculated samples were then cultured aseptically at 16°C for 7 days. Finally, the obtained colonies were observed, and only white colonies were selected. Fungal colonies were subcultured and repeated several times until completely bacteria-free. Stock cultures of pure fungal strains were maintained at a temperature of -20°C, using 10% glycerol (v/v) as a preservative.

### Macroscopic and microscopic examination of the fungal isolates

The fungal morphology was macroscopically studied by observing the color, shape, size, and hyphae colony features. For microscopic examination, the slide culture technique and staining with lactophenol cotton blue along with fungal culture on SDA for 5 - 7 days were performed (15).

### Fungal identification

The fungal isolates’ strain-specificity was confirmed using the method employed by Cao et al. (16). The method involved extracting total genomic DNA from the mycelium and isolating the nuclear ribosomal DNA, including ITS1-5.8S-ITS2, using DreamTaq DNA polymerase and primers ITS4 and ITS5. The PCR amplification was done using a thermal cycler, and the resulting products were analyzed via agarose gel electrophoresis, purified, and sequenced for comparison against the GenBank data set.

### Mycelial biomass production

A new fungus of *O. sinensis* OS8 was applied in this investigation. The fungal culture was preserved on PDA slants at a temperature of 4°C, after which it was regularly sub-cultured every four weeks. The submerged culture was used for the biomass production of *O. sinensis* OS8 using a 24-oz culture bottle. Submerged culture media of 30 ml/bottle was used. The media composition (g/l) by Leung et al. (9) included 200 g of potato, 40 g of glucose (Merck, Germany), 10 g of yeast extract (Becton, USA), 5 g of peptone (Becton, USA), 1 g of KH_2_PO_4_ (Merck, Germany), and 0.5 g of MgSO_4_ (Ajax, Australia). The stock mycelial inoculum of 1x1 cm size in PDA was used and placed in the center of the culture bottle. The culture was maintained in darkness at 16°C for 14 days. Afterward, the mycelial biomass was centrifuged at 3,000 *g* for 15 min, washed with sterile water, filtered, and then dried at 60°C for 24 h in a hot air oven.

### Mycelial biomass adenosine extraction

The extraction methods described by Huang et al. (17) were utilized to extract adenosine from *O. sinensis* OS8 mycelia, as follows: the mycelia of *O. sinensis* OS8, which had been dried, were pulverized into a powder using liquid nitrogen. Approximately 1.0 g of the powder was combined with 10 ml of a 50/50 (v/v) mixture of ethanol and water in a 50 ml centrifuge tube. The samples were subsequently placed in an ultrasonic machine to extract adenosine at a power of 75 W at 50°C for 60 min. The sample extraction procedures were repeated twice. Prior to HPLC analysis, the samples’ supernatants were filtered using a 0.45 µm filter. The investigations were performed in triplicate.

### Determination of adenosine

HPLC (Agilent, HP 1100 series, USA) analysis was conducted with an auto-injector and a reverse phase analytical column (Hypersil ODS C18 4 × 250 mm, 5 µm, UK). The standard of adenosine (Sigma, USA) was injected to generate a calibration curve. The injection volumes were 12.5, 25, 50, 100, and 200 µg/ml. The mobile phase was a mixture of 0.05 M potassium dihydrogen phosphate, water/methanol (85:15, v/v), and water/acetonitrile (95:5, v/v). The conditions for HPLC analysis included an injection volume of 5 µl, and the analysis was performed with a flow rate of 0.5 ml/min, a detection wavelength of 242 nm, and the column temperature maintained at 20°C.

### Mycelial biomass polysaccharide extraction

The extraction methods of Wang et al. (14) were used to extract *O. sinensis* OS8 polysaccharide (OS8P). To prepare the extract, 10 g of mycelium powder was dissolved in 100 ml of distilled water for 2 h, followed by ultrasonic extraction at 50°C for 15 min. The sample was mixed with 300 ml of 95% ethanol, stirred, and settled at 4°C for 18 h. It was then separated via centrifugation at 3,000 *g* for 15 min and the resulting OS8P precipitate was rapidly pre-frozen at -35°C for 1 h before undergoing vacuum freeze-drying. Finally, the obtained OS8P was further investigated for anticancer, antioxidant, and immunomodulatory activities.

### Determining α- and β-D-glucan levels in OS8P extract

The bioactive glucan compounds were quantified using a mixed-linkage β-D-glucan assay kit obtained from Megazyme International (Ireland), as per the method described by McCleary and Draga (18).

### Measurement of total D-glucan and D-glucose in free D-glucose and sucrose

McCleary and Draga’s method (18) was used to determine the total D-glucan and D-glucose in free D-glucose and sucrose. The process involved weighing 10 mg of OS8P sample powder and adding it to each tube containing 2.0 ml of ice-cold 12 M sulfuric acid. The tubes were then placed in an ice-water bath for 2 h before adding distilled water, boiling in a water bath, and incubating for 2 h. The contents were transferred to a 100 ml volumetric flask, mixed, and centrifuged. Then, 0.1 ml of sample solution was mixed with exo-1,3-β-glucanase and β-glucosidase and incubated at 40°C for 60 min. GOPOD reagent was added, and the absorbance was measured at 510 nm.

### Quantifying α-D-glucan and D-glucose in the presence of phytoglycogen, starch, sucrose, and free D-glucose

To begin the process (18), 10 mg of OS8P powdered sample was mixed with 2 ml of 1.7 M NaOH in a tube and stirred in an ice-water bath for 20 min. Then, 8 ml of 1.2 M sodium acetate buffer (pH 3.8) was added to each tube with stirring, followed immediately by the addition of 0.2 ml of amyloglucosidase (1,630 U/ml) and invertase (500 U/ml). The tubes were then incubated in a 40°C water bath for 30 min. Samples containing less than 10% α-glucan content were centrifuged at 3,000 *g* for 5 min, and 0.1 ml of the resulting solution was mixed with 0.1 ml of sodium acetate buffer (200 mM, pH 4.5) and 3.0 ml of GOPOD reagent. The tube contents were incubated for 20 min at 40°C before measuring the absorbance at 510 nm against the blank reagent. The calculation for glucan is as follows:


Total D−glucan (% w/w)=ΔA × F/W × 90

α−D−glucan (% w/w)=ΔA × F/W × 9.27 (final volume 10.3 ml)

β−D−glucan=Total D−glucan−α−D−glucan

where: ΔA = reaction absorbance – blank absorbance.

F = a factor to convert absorbance to μg of D-glucose. = 100 (μg of the D-glucose standard) GOPOD absorbance for 100 μg of D-glucose standard.

W = weight of sample.

### OS8P bioactive compound analyses by GC-MS

The chemical components of OS8P were analyzed using a gas chromatograph with mass spectrometry (GC-MS), following the modified method of Chit-aree et al. (19). Sample preparation involved mixing 0.1 mg of OS8P with 1 ml of anhydrous ether (Merck, Germany). GC-MS analysis was conducted using an Agilent HP 5890 gas chromatography instrument coupled with an Agilent MSD HP 5970 mass selective detector and HP 59970 MS Chemstation with a 59973 NBS mass spectral library (NBS_REVF). A fused silica column of 25 m × 0.20 mm × 0.25 µm film thickness (Varian Inc., Agilent Technologies, USA) was used for separation, and each extracted sample of 1 µl was injected in an injector operation. High-purity helium gas (99.999%) was used as a carrier gas at a 1.0 ml/min flow rate. The ion source and quadrupole mass analyzer were kept at 230 and 150°C, respectively, and the analysis was conducted for 60 min per sample. The MSD Chemstation software (Agilent Technologies) and commercial libraries (NIST02.L, NIST05.L, and Wiley7Nist05.L) were used to identify the chemical compounds of OS8P.

### Proliferative inhibition of colon cancer cells: cell line culture

HT-29 human colon cancer cells (ATCC) were cultured in a 25 cm^3^ flask (Nunc, Denmark) with Dulbecco’s Modified Eagle Medium (DMEM) (Gibco, USA) containing 10% fetal bovine serum (FBS) (Gibco, USA), 1% non-essential amino acid (Gibco, USA), and 1% penicillin-streptomycin (Gibco, USA). The cells were incubated at 37°C under a 5% CO_2_ atmosphere in a CO_2_ incubator (Forma Scientific, 3111, USA) and subcultured after trypsinization once they reached confluence. These colon cancer cells were used for subsequent investigations of antiproliferation and apoptosis.

### Cell survival assay

The MTT assay (20, 21) was utilized to evaluate cell survival. Cells (100 µl at 10^6^ cells/ml density in DMEM medium) were seeded in a 96-well plate (Nunc, Denmark), incubated for 24 h, washed, and exposed to different concentrations of OS8P (0, 25, 50, 100, 200, and 400 µg/ml) for 48 h. Following incubation, the cells were washed twice with PBS, and MTT (Sigma, USA) solution (0.5 mg MTT/ml in DMSO) was added to each well. The plate was then incubated in the dark at 37°C for 4 h, and absorbance was measured at 595 nm using a microplate reader after solubilizing the formazan precipitates with DMSO (Sigma, USA). This experiment was repeated three times with eight replicates per observation, and the inhibition rate of cell proliferation was calculated.


$$\text{Antiproliferation}\left(\%\right)=100\;\times \;\left[1\;–\left({\text{OD}}_{\text{sample}}/{\text{OD}}_{\text{control}}\right)\right]$$

### Apoptotic induction by cell morphological stained

#### AO/PI double staining

To measure the OS8P-induced cell death in HT-29 cells, acridine orange/propidium iodide (AO/PI) (Sigma-Aldrich) double staining (22) was utilized. The treatments were conducted in an 8-well culture slide (Nunc, Denmark), where 400 µl of HT-29 cells (1 × 10^6^ cells/ml) was treated with 400 µl of OS8P (200 µg/ml). Following that, the cells were subjected to incubation at 37°C in a 5% CO_2_ atmosphere for a duration of 48 h. Subsequently, the supernatant was discarded, and the remaining media was eliminated by washing the cells twice with PBS. Subsequently, 300 µl of fluorescent dyes containing AO (10 µg/ml) and PI (10 µg/ml) was added into the well, and the cells were incubated for 5 min in the dark. After the incubation, the supernatant was discarded, and a fluorescent microscope (Olympus, BX51, Japan) was used to visualize the apoptotic and live cells. This experiment was accomplished in triplicate, and three independent studies were performed for every single treatment.

#### DAPI staining

The DAPI method [modified from (23)] was used to evaluate nuclear changes in apoptotic cells. HT-29 cells were seeded in an 8-well culture slide (Nunc, Denmark) and incubated for 24 h. Next, 400 µl of OS8P (200 µg/ml) was added to 400 µl of HT-29 cells (1.2 × 10^4^ cells/ml), and the cells were incubated for 48 h. After washing twice with PBS, 300 µl of DAPI fluorescent dye (100 µg/ml) was added and incubated for 15 min. A fluorescent microscope (Olympus, BX51, Japan) was used to visualize apoptotic and live cells. The experiment was done in triplicate, and three independent studies were conducted for each treatment.

### DNA fragmentation

DNA fragmentation in HT-29 cells treated with OS8P at 200 µg/ml was assessed using a modified method based on Wasunan et al. (24). Cells were seeded in 6-well plates, treated with either 1 nM Paclitaxel or 200 µg/ml of OS8P, and incubated for 48 h. Genomic DNA was extracted, and electrophoresis was performed on a 1.5% agarose gel for 45 minutes at 80 V.

### Morphological changes in colon cancer cells using scanning electron microscopy (SEM)

HT-29 colon cancer cells were cultured in DMEM supplemented with FBS and antibiotics, and were incubated under standard conditions. After attaching to 24-well plates, the cells were treated with OS8P at its IC_50_ concentration for 24 h. The fixed cells were washed and dehydrated with a series of ethanol solutions, then coated with gold for scanning electron microscopy (SEM; HV SEM; TESCAN, Vega III, Czech Republic) imaging, following previous protocols (25).

### Antioxidant activity and determination of DPPH radical scavenging activity of OS8P

The scavenging activity of OS8P against DPPH radical was determined using a modified version of the procedure by Wang et al. (26). Different concentrations (0.1-0.7 mg/ml) of OS8P solution were mixed with DPPH-methanol solution and incubated for 30 minutes. Absorbance was measured at 517 nm using a microplate reader, and positive controls of α-tocopherol and Trolox were used as reference standards. The scavenging percentage was calculated using the equation:


Scavenging activity(%)=[1− (Aa−Ab)/Ac]×100

where the absorbance of the DPPH reagent in the occurrence of the solutions is represented as Aa, the absorbance of the solutions is represented as Ab, and the absorbance of the DPPH as the blank control in the absence of the solutions is represented as Ac.

### Assessment of ABTS radical cation scavenging activity

The method in this study was modified from Xiao et al. (27). To prepare ABTS•+ (Sigma, USA), 20 ml of 7.4 mM ABTS solution was mixed with 13.2 mg K_2_S_2_O_8_. Then, 150 µl of OS8P solution at different concentrations (0.10 to 1.25 mg/ml) was added to 50 µl of the diluted ABTS•+ solution in a 96-well plate, which was kept in the dark for 6 min. Absorbance was measured at 734 nm using a microplate reader. Positive controls, such as α-tocopherol and trolox, were used. The ability of OS8P to scavenge ABTS•+ was calculated using the following equation:


$${\text{ABTS}}^{•+}\text{scavenging ability}\left(\%\right)=\left[1-\;\left(\text{Aa}-\text{Ab}\right)/\text{Ac}\right]\times 100$$

when the absorbance of the solutions is denoted as Ab, the absorbance of ABTS•+ in the presence of the solutions and the absorbance of ABTS•+ as the blank control in the absence of the solutions are denoted respectively as Aa and Ac.

### Cell immune stimulation

#### Animals and splenocyte preparation

In this study, 6-week-old male Wistar Hannover GALAS rats were used. The use of animals in our investigation was reviewed and approved by Maejo University (MACUC 033S/2564). The method employed by Srikham and Thirabunyanon (28) was used. The spleens were removed aseptically from the rats, which were killed by cervical dislocation. The spleen tissue was homogenized and filtered through 70 µm cell strainers to obtain a single-cell suspension of splenocytes. After washing with PBS, the viability of the cells was checked using trypan blue exclusion. The cells were then adjusted to a concentration of 1.5 × 10^6^ cells/ml in RPMI 1640 (SPL Life Science, Korea) medium.

### Cell immune proliferation

The MTT colorimetric method was used to determine spleen cell proliferation, following the protocol by Lee et al. (29). Spleen cell suspensions at 2.0 × 10^5^ cells/ml were added to 96-well plates with 50 µl of B or T lymphocyte activator (LPS or ConA, 10 µg/ml) and 50 µl of OS8P at various concentrations (0 - 200 µg/ml). After 72 h of incubation, MTT (5 mg/ml) was added to each well and incubated for 4 h. Then, formazan crystals were dissolved in DMSO, and the absorbance at 595 nm was measured using a microplate reader (20).

### Statistical analysis

The mean value with standard deviation was used to present the results, which were analyzed using IBM SPSS. Multiple comparisons between groups were conducted with one-way ANOVA and Duncan’s multiple range test. A significance level of *P*< 0.05 was considered statistically significant.

## Results

### Mycelial isolation from dried *O. sinensis* fruiting bodies

The process of isolating fungal mycelia from *O. sinensis* fruiting bodies involves a series of steps that are crucial in ensuring successful fungal growth. The use of dried fruiting bodies is a common method of extracting mycelia since it reduces the risk of contamination by other microorganisms. The fact that most of the dried *O. sinensis* fruiting bodies did not exhibit any mycelial growth suggests that the isolation process is highly dependent on a number of factors, such as the quality of the fruiting bodies, environmental conditions, and the techniques employed during isolation. This highlights the importance of standardizing the isolation process to ensure consistency in the results obtained. The 14 dried *O. sinensis* fruiting bodies that showed positive signs of fungal mycelia development were found to have distinct morphologies, such as a white or cream center. These variations in morphology may be attributed to differences in the genetic makeup of the fungal mycelia, which can impact their growth characteristics.

The successful cultivation of these mycelial colonies is a significant step towards further analysis and understanding of their properties. These properties may include bioactive compounds, which are of great interest in pharmaceutical and medicinal research. However, it is important to note that the results obtained from this study are specific to the *O. sinensis* species and may not necessarily apply to other fungal species. The isolation of fungal mycelia from *O. sinensis* fruiting bodies is a crucial step towards further analysis and understanding of their properties. The process is highly dependent on several factors, and standardization is necessary to ensure consistency in results. The distinct morphologies exhibited by the mycelial colonies provide valuable insight into their genetic makeup and growth characteristics, which may have important implications in various fields, including medicine and biotechnology.

### Fungal identification and mycelial morphology

Identifying fungal genera is a complex task that requires thorough analysis of various species. In this study, **Table 1** presents the different species used to identify the genera of fungi. The most frequently identified genus was *Penicillium roqueforti*, while *Nigrospora oryzae* had a lower prevalence but has been found to produce bioactive compounds. The remaining genera, including *Penicillium soppii*, *Megasporoporia minor*, *Ophiocordyceps sinensis*, and *Truncatella angustata*, were identified in only one colony each, suggesting their potential uniqueness. Further studies are needed to fully understand their properties and benefits.

**Table 1:** The fungal strains of mycelial colonies isolated from natural Ophiocordyceps sinensis fruiting bodies.

Isolate Specimen code	Fungal species	Accession no. of GenBank	Identity (%)
OS1	*Penicillium soppii*	MH865750.1	97.93
OS2	*Megasporoporia minor*	MN398984.1	99.68
OS3	*Penicillium roqueforti*	MH858924.1	99.65
OS4	*Truncatella angustata*	MT514384.1	99.83
OS5	*Penicillium roqueforti*	MH858924.1	99.48
OS6	*Penicillium roqueforti*	KY315937.1	99.48
OS7	*Penicillium roqueforti*	MH858924.1	99.65
OS8	*Ophiocordyceps sinensis*	KX237742.1	98.40
OS9	*Penicillium roqueforti*	MH858967.1	100.00
OS10	*Penicillium roqueforti*	MH858924.1	99.83
OS11	*Penicillium roqueforti*	KX100382.1	100.00
OS12	*Penicillium roqueforti*	MH855127.1	99.83
OS13	*Nigrospora oryzae*	MZ882151.1	99.81
OS14	*Nigrospora oryzae*	MH748173.1	100.00

Moreover, *O. sinensis* OS8 was investigated against several aspects, including its morphological characteristics. A colony of *O. sinensis* OS8 exhibited white, plumose, downy, and dense aerial mycelium and hyphal strands, with interwoven hyphae, rod-shaped spores, and were mostly 5 – 10 µm long. *O. sinensis* is a well-known medical mushroom used in traditional medicine, highlighting the potential for further investigation of *O. sinensis* OS8 in various fields.

### Mycelial biomass production

In this investigation, the *O. sinensis* OS8 strain was chosen as the source for biomass production, due to its potential for high yields. The biomass obtained from this strain was then subjected to a polysaccharide extraction, followed by an examination of its bioactivities. To obtain the mycelial biomass, *O. sinensis* OS8 was cultured in liquid media for a period of 14 days. The resulting mycelial biomass was then harvested and dried, yielding a total of 23.61 g/l of dried weight, as shown in **Table 2**. The high yield of mycelial biomass obtained from *O. sinensis* OS8 is a promising indication of its potential as a source of bioactive compounds.

**Table 2:** The mycelial biomass production yield, adenosine content, and mycelial biomass polysaccharide extraction yield.

	Mycelium biomass (g/l) (mean ± SD, n = 100)	Adenosine content (mg/100 g) (mean ± SD, n = 3)	Mycelial biomass polysaccharide (g/100 g) (mean ± SD, n = 10)
Yields	23.61 ± 2.01	306.10 ± 1.31	3.22 ± 0.015

### Mycelial biomass adenosine composition

The dried mycelial biomasses of *O. sinensis* OS8 were analyzed using HPLC to determine the adenosine compound present. The results showed a significant adenosine content of 306.1 mg per 100 g of dried mycelial biomass (**Table 2**).

### Mycelial biomass polysaccharide extraction

Polysaccharide extraction from dried mycelial biomass of *O. sinensis* OS8 was further performed using ultrasonic instrument. As a result, obtained polysaccharide yield was at the rate of 3.22 g/100 g of dried weight (**Table 2**).

### Bioactive D-glucan, α-, and β-D-glucan compositions of biomass mycelial polysaccharides

**Figure 1:**
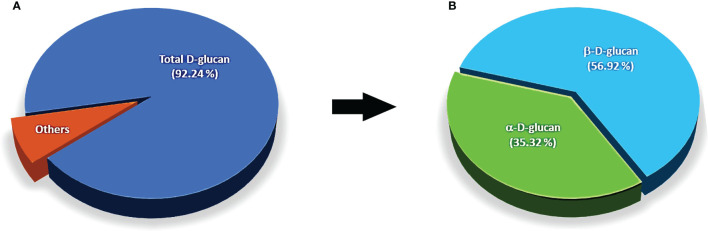
The ratios of polysaccharide compositions as D-glucan **(A)**, α-, and β-D-glucan **(B)** from dried mycelial biomass of *O. sinensis* OS8.

The ratios of polysaccharide compositions as D-glucan, α-, and β-D-glucan from dried mycelial biomass of *O. sinensis* OS8 polysaccharides (OS8P) are shown in **Figure 1**. Of our results, with the new findings, it showed that these polysaccharides of OS8P were enriching β-D-glucan at a percentage of 56.92% (**Figure 1B**). Also, another type of α-D-glucan was found at a percentage of 35.32% (**Figure 1B**). The total polysaccharide D-glucan in the OS8P was obtained at a rate of 92.24% (**Figure 1A**).

### Chemical composition of biomass mycelial polysaccharide

The GC-MS analysis identified the compositions of OS8P, as shown in **Table 3**. The main components of OS8P were dodecamethyl pentasiloxane, 2,6-bis (methylthiomethyl) pyridine, 2-(4-pyrimidinyl)-1H-Benzimidazole, and 2-Chloro-4-(4-nitroanilino)-6-(O-toluidino)-1,3,5-triazine at the rates of 32.5, 20.0, 17.5, and 16.25%, respectively.

**Table 3:** Chemical composition of the OS8P (mean ± SD, n = 3).

No.	Rt (min)	Compound	formula	%
1	16.60	Dodecamethyl pentasiloxane	C_12_H_36_O_4_Si_5_	32.50 ± 0.10
2	19.02	2-Chloro-4-(4-nitroanilino)-6-(O-toluidino)-1,3,5-triazine	C_5_H_6_ClN_3_O_2_	16.25 ± 0.01
3	21.83	2,6-bis (methylthiomethyl) pyridine	C_9_H_13_NS_2_	20.00 ± 0.00
4	23.22	Tetrasiloxane	H_10_O_3_Si_4_	03.75 ± 0.02
5	25.09	2-(4-pyrimidinyl)-1H-Benzimidazole	C_11_H_8_N_4_	17.50 ± 0.10
6	29.86	N-(2-Chloro-1-ethoxyethyl)-N-cyano-N’,N’,N’’,N’’-tetramethyl-1,3,5-triazine-2,4,6-triamine	C_12_H_20_ClN_7_O	03.75 ± 0.01
7	40.39	Decamethyl tetrasiloxane	C_10_H_30_O_3_Si_4_	02.50 ± 0.00
8	42.19	N-ethyl-1,3-dithioisoindoline	C_10_H_13_NS_2_	01.25 ± 0.01
9	44.79	Hexamethyl cyclotrisiloxane	C_6_H_18_O_3_Si_3_	02.50 ± 0.00

Cut-off value of the NIST MS library matching was set at 85 (%).

### Colon cancer cell antiproliferation

**Figure 2:**
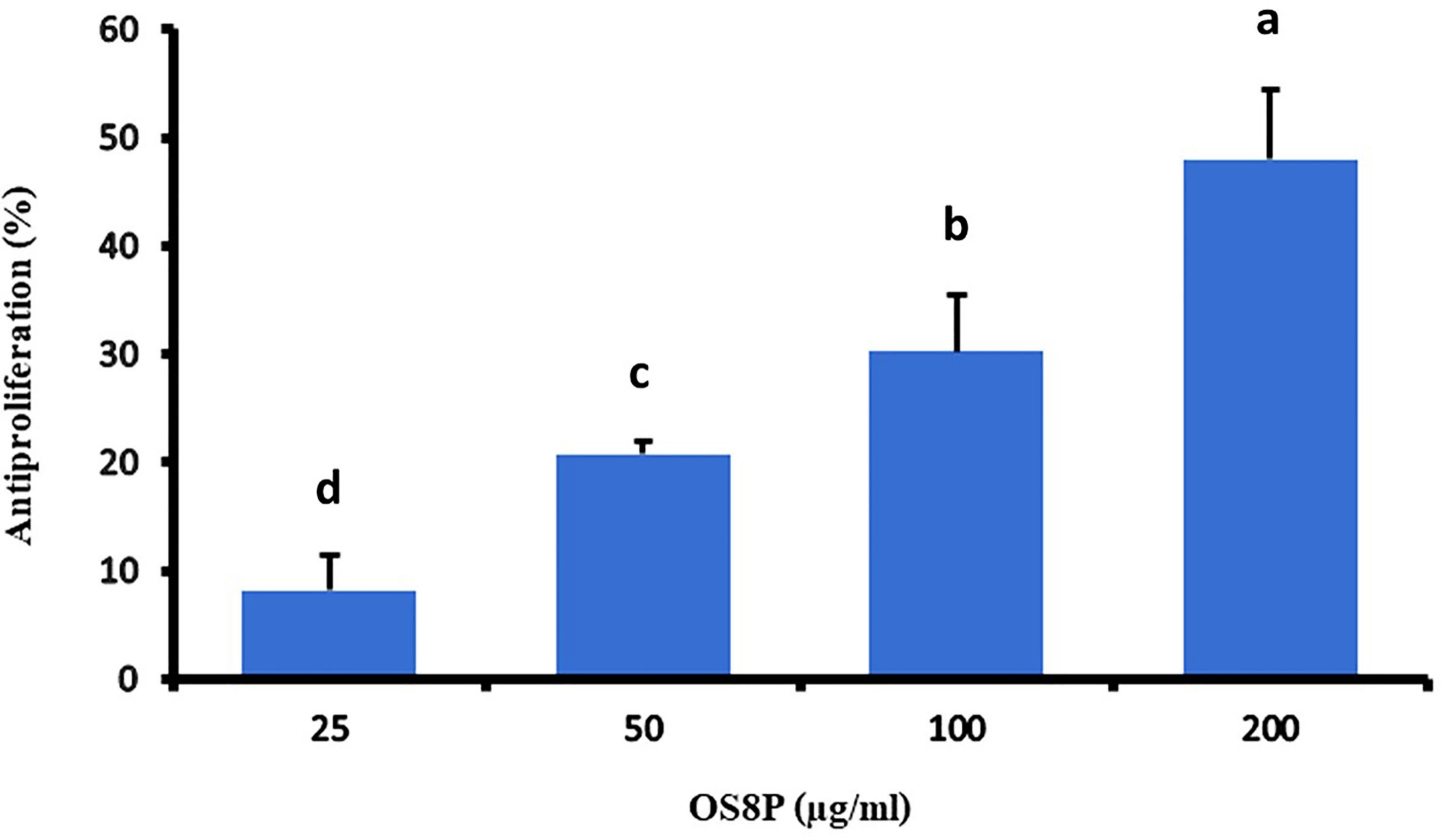
Inhibitory effects of the OS8P on the growth of HT-29 cells. All data are expressed as mean ± SD of three independent experiments. Different letters above the error bars indicate statistically significant differences between groups (*P*< 0.05).

This study investigated the potential action of OS8P on anticancer activity against colon cancer. OS8P challenge with various concentrations of 25, 50, 100, and 200 µg/ml in colon cancer cells resulted in a dose-dependent concentration on inhibition of colon cancer cell proliferation. In this growth inhibition, all concentrations of OS8P significantly inhibited HT-29 cells (*P*< 0.05) compared to the control group. The growth inhibition of colon cancer cells at the concentration of OS8P with 25, 50, 100, and 200 µg/ml were 8.20, 20.81, 30.23, and 48.00%, respectively (**Figure 2**). The IC_50_ value of OS8P efficacy on antiproliferation of colon cancer cells was 202.98 µg/ml.

### Apoptotic induction

**Figure 3:**
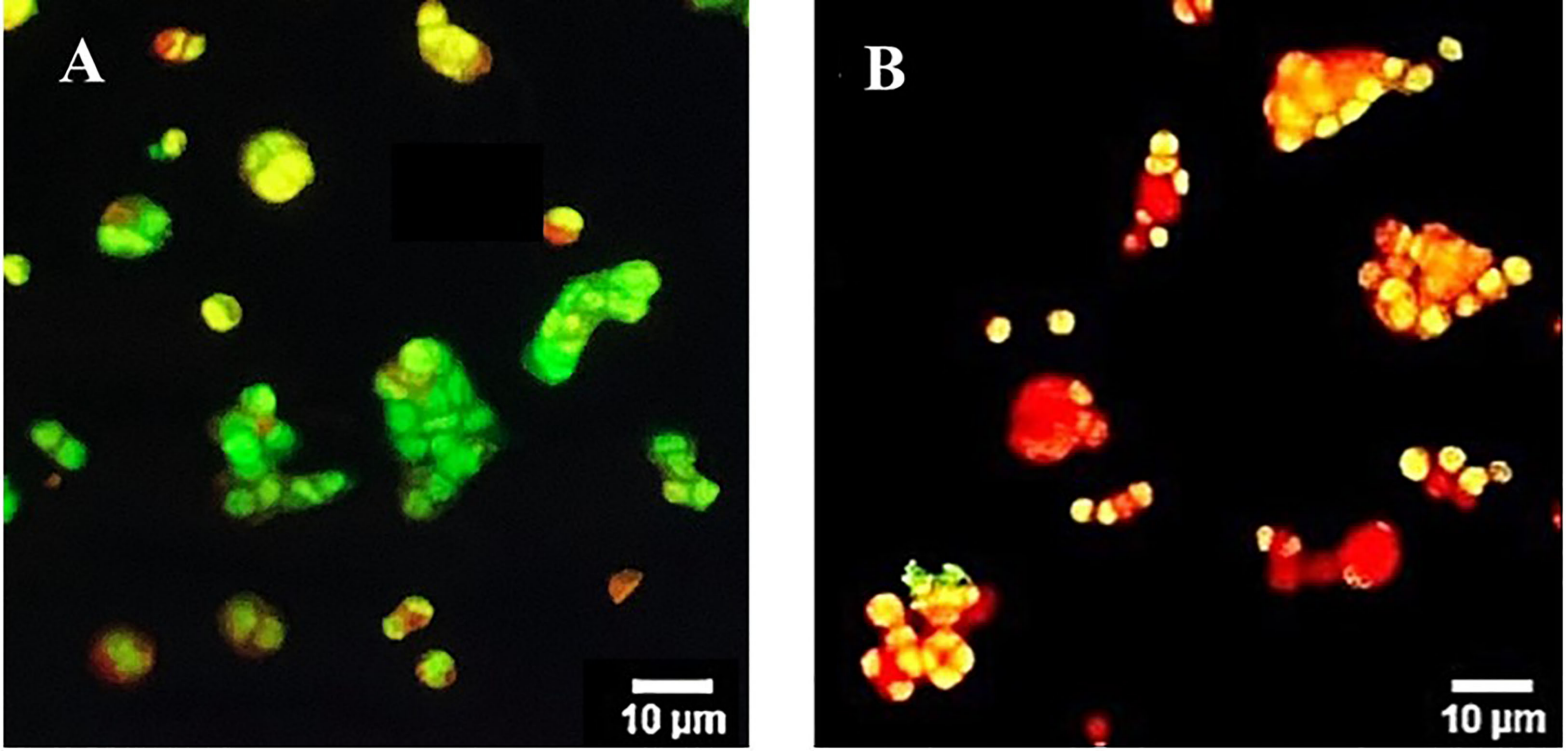
Morphological changes of HT-29 cells using AO/PI staining in combination and visualized via fluorescence microscopy. Cells were treated with **(B)** OS8P (200 µg/ml), and **(A)** non-treated OS8P as control; green fluorescence indicates normal cells while orange/red fluorescence indicates apoptotic cells.

The apoptotic involvement in the OS8P-induced colon cancer cell death was further investigated. The cell apoptosis could be distinguished by simultaneously staining with AO/PI and visualized by fluorescence microscope. The morphology change of the OS8P-treated HT-29 cells differed from those of the untreated cells. The cell membrane and nucleus of treated cells were stained orange or red, indicating that they were apoptotic-induced (**Figure 3B**), whereas the control cells were not (**Figure 3A**).

**Figure 4:**
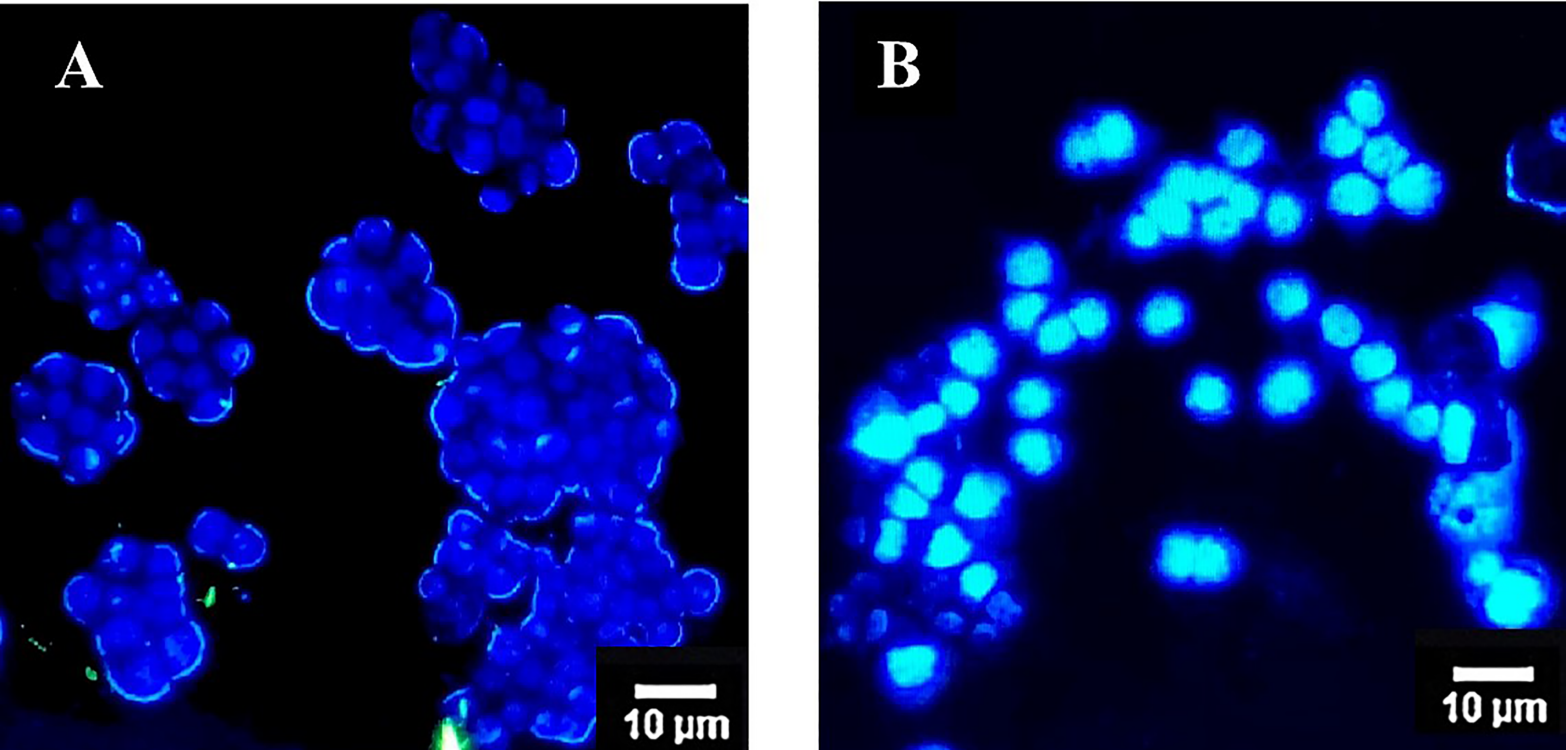
Morphological changes of HT-29 cells using DAPI staining and visualized via fluorescence microscopy. Cells were treated with **(B)** OS8P (200 µg/ml), and **(A)** non-treated OS8P as control; blue non-fluorescence indicates normal cells while blue fluorescence indicates apoptotic cells.

Exposure of the colon cancer cells with OS8P for 24 h resulted in apoptotic effects in HT-29 cells after being visualized by a fluorescence microscope. Cell morphological change of the OS8P-treated HT-29 cells was different from the untreated cells. A blue glow of the OS8P-treated cell nucleus appeared under fluorescence microscopy, which indicated that they were apoptotic-induced (**Figure 4B**), whereas the control cells were blue non-glow (**Figure 4A**).

### DNA fragmentation

**Figure 5:**
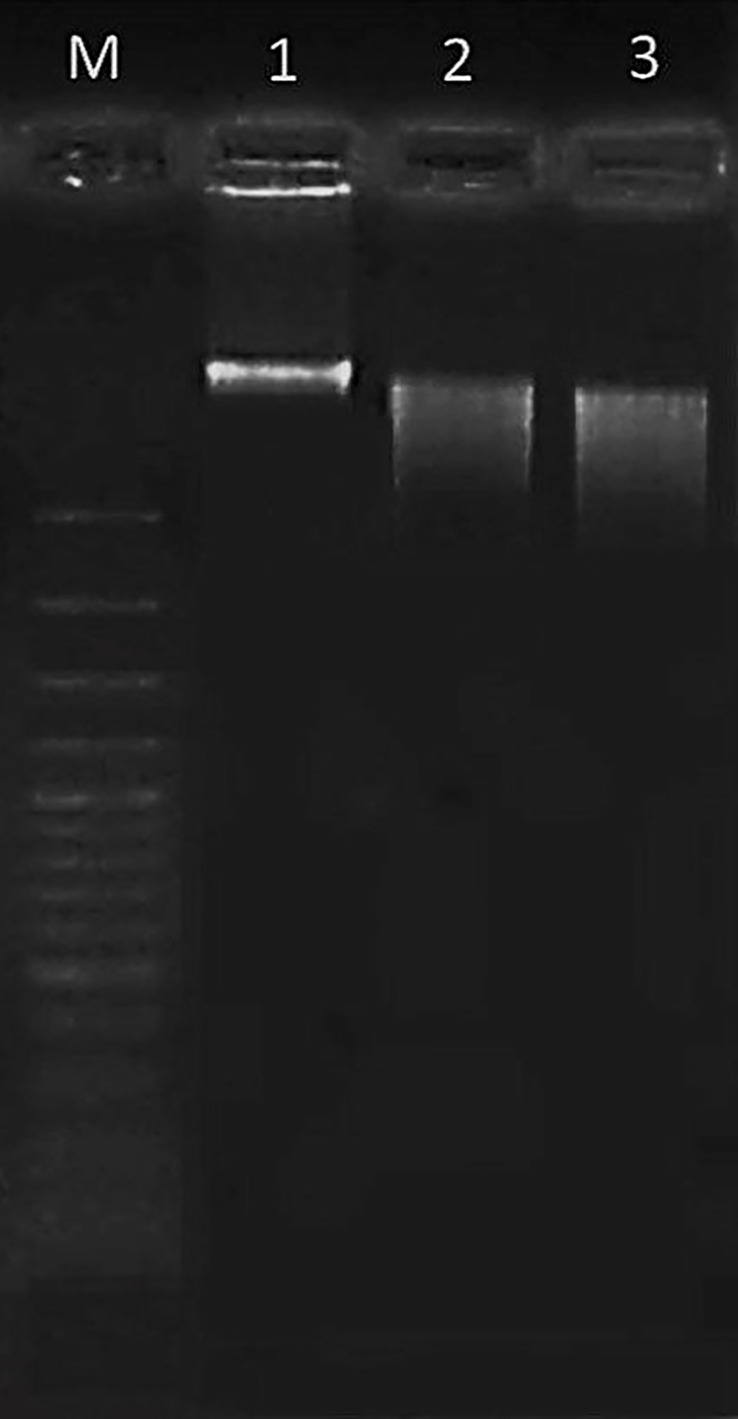
DNA fragmentation of HT-29 cells treated with OS8P (200 µg/ml). Lane M; DNA ladder, lane 1; control, lane 2; Paclitaxel as positive control, and lane 3; OS8P. The living cells (control group) showed non-fragmented DNA. The cell death apoptosis (Paclitaxel and OS8P) was characterized by the small DNA fragments.

The occurrence of cell death apoptosis in colon cancer cells was seen through DNA fragmentation, as shown in **Figure 5**. The non-fragmented DNA appeared in the untreated HT-29 cells (lane 1), as indicated in living cells. After being challenged with Paclitaxel or OS8P for 48 h, the small DNA fragments occurred in both groups of Paclitaxel (positive control, lane 2) or OS8P (lane 3), as evidenced in colon cancer cell death apoptosis.

### Morphological changes of colon cancer cells by SEM

**Figure 6:**
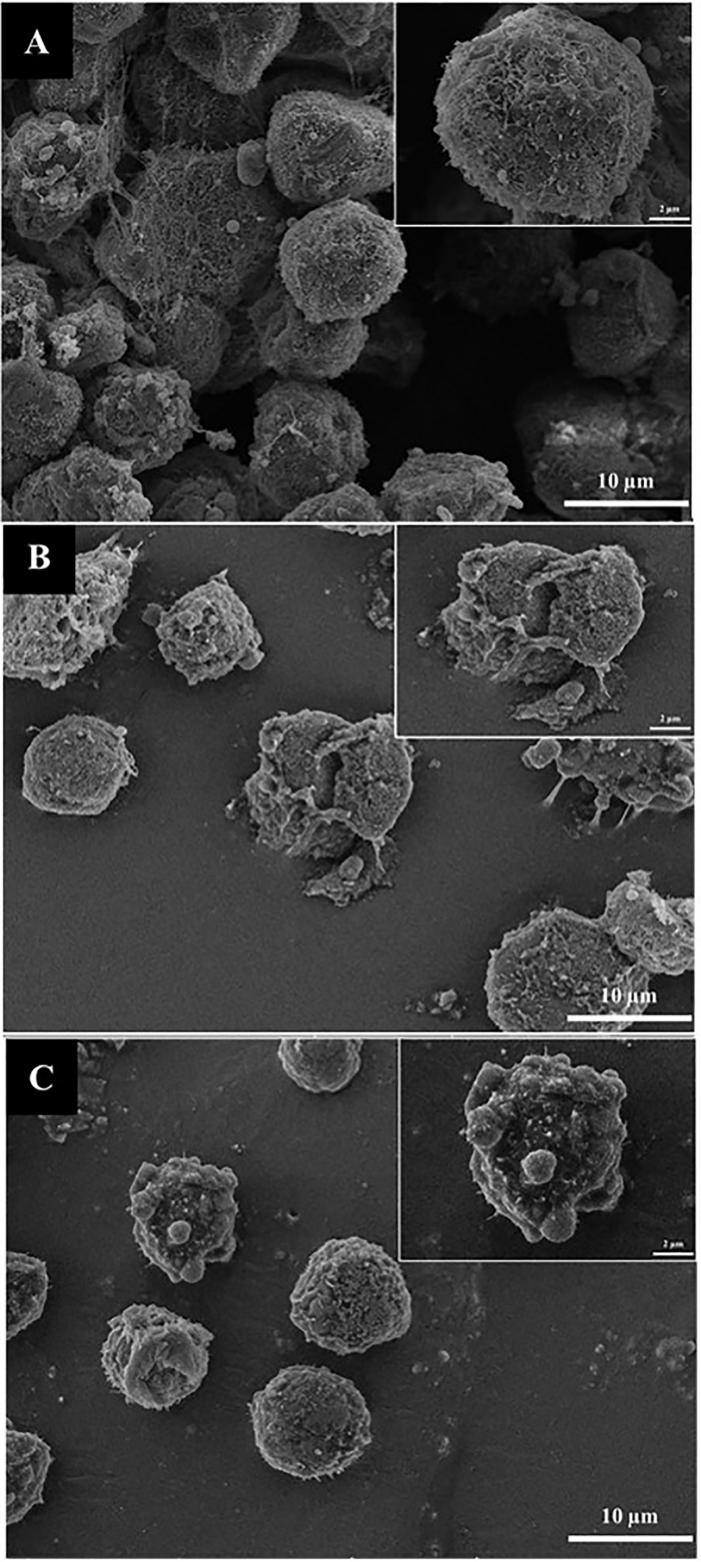
Morphological changes of HT-29 cells visualized under SEM. Cells were treated with **(C)** OS8P (200 µg/ml), **(A)** non-treated OS8P as control; and **(B)** Paclitaxel as positive control. The living cells (control group) showed spherical shape, numerous microvilli on surface, and were generally kept intact. The cell death apoptosis (OS8P and Paclitaxel) was characterized by cell lysis, blebbing formation, loss of microvilli, and appearance of apoptotic bodies.

The morphological changes of colon cancer cells induced by OS8P, HT-29 cell deaths were observed under SEM. As illustrated in **Figure 6**, the cells in the control group showed a spherical shape, numerous microvilli on the cell surface, and were generally kept intact (**Figure 6A**). At the same time, morphological changes of apoptotic cells characterized by cell lysis, loss of microvilli, blebbing formation, and appearance of apoptotic bodies were found after treatment with Paclitaxel (**Figure 6B**) as positive control and challenged with 200 µg/ml OS8P (**Figure 6C**).

### Antioxidant activity

**Figure 7:**
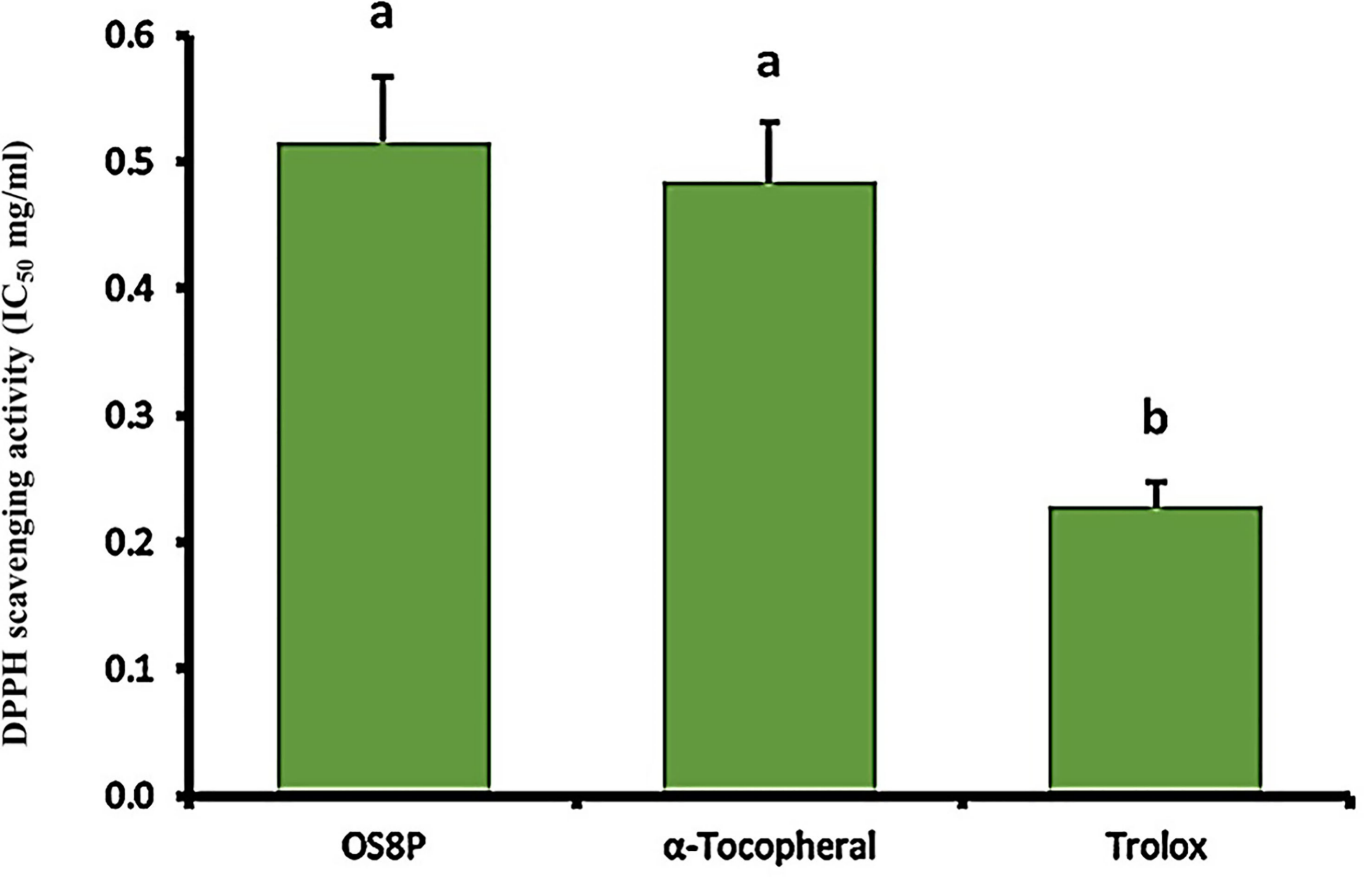
The DPPH scavenging activity of OS8P. All data are expressed as mean ± SD of three independent experiments. Different letters above the error bars indicate statistically significant differences between groups (*P*< 0.05).

The DPPH and ABTS aspects were performed in this study. A DPPH scavenging activity of OS8P was at the IC_50_ value of 0.52 mg/ml, which was not different with α-tocopherol as a positive control at the 0.48 mg/ml rate. However, the IC_50_ value of Trolox as another positive control at the rate of 0.23 mg/ml was significantly lower (*P*< 0.05) than that of OS8P (**Figure 7**).

**Figure 8:**
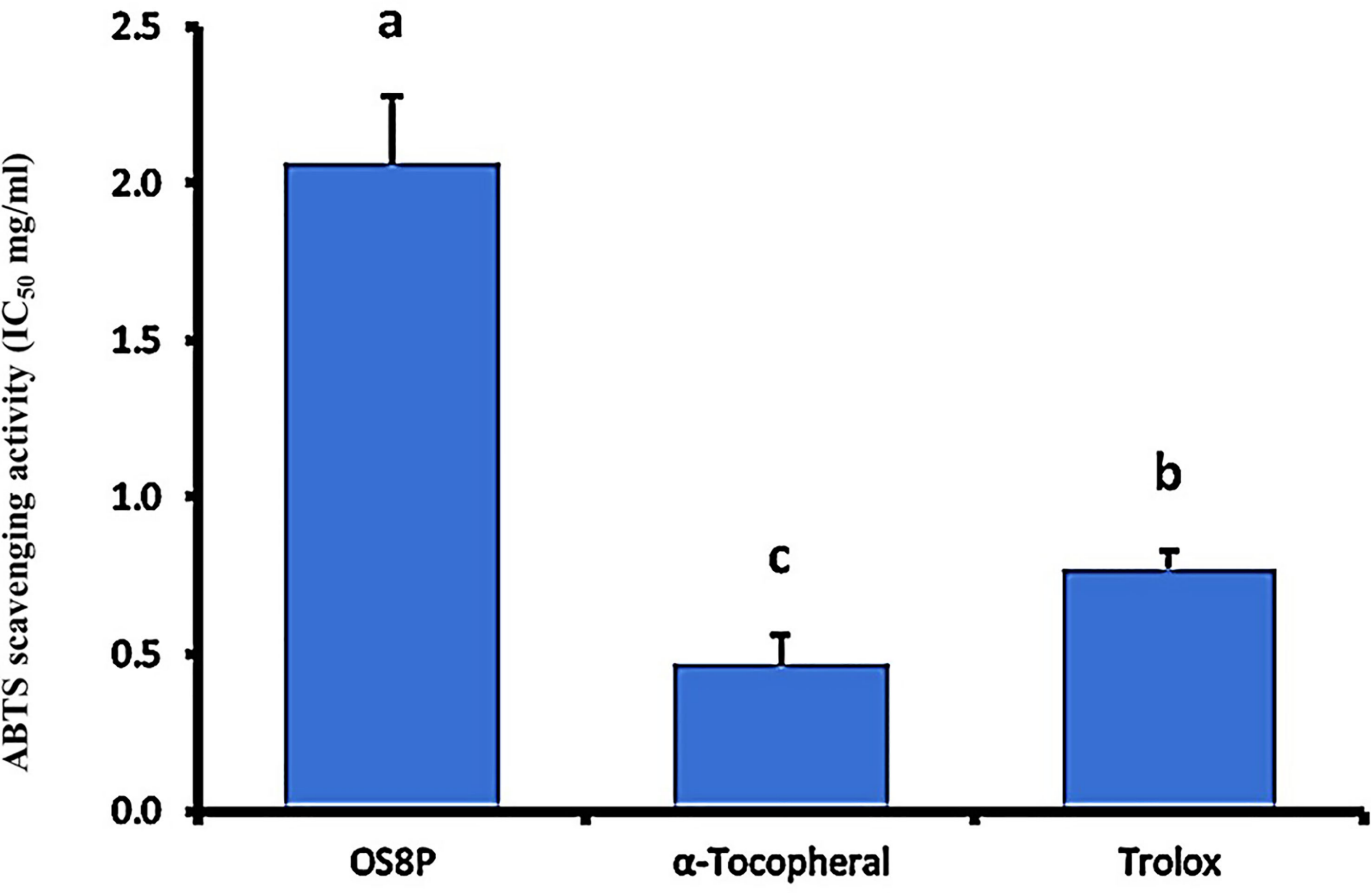
The ABTS radical scavenging activity of OS8P. All data are expressed as mean ± SD of three independent experiments. Different letters above the error bars indicate statistically significant differences between groups (*P<* 0.05).

To correlate to the ABTS scavenging activity, the antioxidant property was found in the OS8P with the IC_50_ value of 2.07 mg/ml. However, it was significantly higher (*P*< 0.05) as compared to the positive controls of α-tocopherol and trolox at the rates of 0.47 and 0.77 mg/ml, respectively (**Figure 8**).

### Cell immune activation

**Figure 9:**
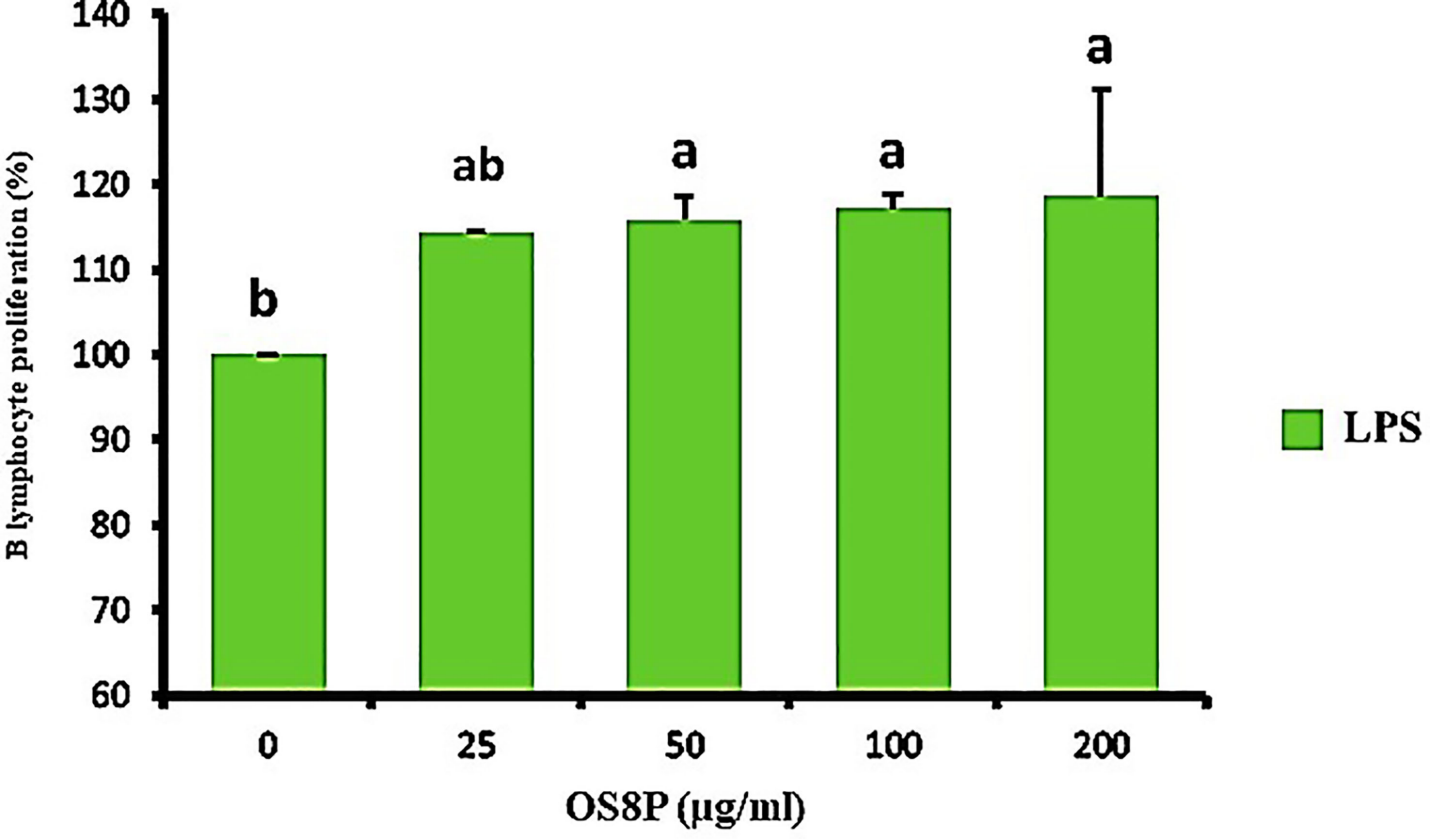
Lymphocyte proliferation via the OS8P and LPS stimulation. All data are expressed as mean ± SD of three independent experiments. Different letters above the error bars indicate statistically significant differences between groups (*P*< 0.05).

**Figure 10:**
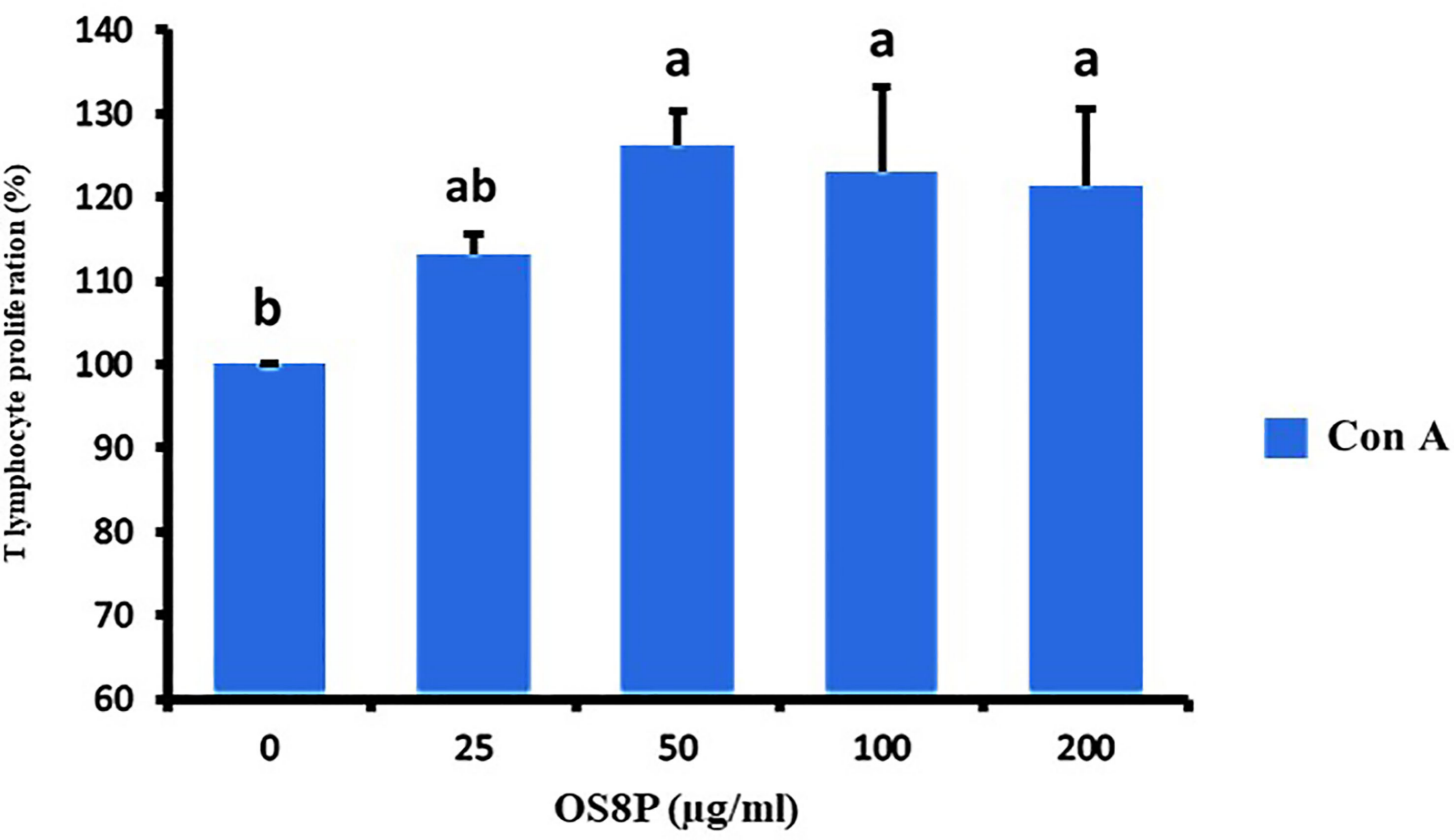
Lymphocyte proliferation via the OS8P and Con A stimulation. All data are expressed as mean ± SD of three independent experiments. Different letters above the error bars indicate statistically significant differences between groups (*P*< 0.05).

Cell immune induction of rat spleen lymphocyte cell proliferation treated with OS8P is shown in **Figures 9**, **10**. The OS8P induced the spleen cell proliferation with the dose responses and were significantly higher (*P*< 0.05) than the control group of LPS (B lymphocyte cell stimulation) alone, except for the low concentration of OS8P at 25 µg/ml. These proliferation rates of spleen cells in the concentration of 25, 50, 100, and 200 µg/ml were 114.3, 115.8, 117.2, and 118.6%, respectively (**Figure 9**).

The spleen cells were also treated with OS8P and restimulated by ConA (T lymphocyte cell stimulation) in a dose-dependent concentration. It showed a significant increase in splenocyte proliferation (*P*< 0.05) in only high doses of 50, 100, and 200 µg/ml compared to spleen cells treated with ConA alone as the control group. The growth rates of spleen cells in the concentration of 25, 50, 100, and 200 µg/ml were 113.1, 126.1, 123.0, and 121.4%, respectively (**Figure 10**).

## Discussion

There is high demand for rare natural *O. sinensis* in Asia. This is due to low annual productivity and overharvesting. The low production rate each year is related to the long growth period of *O. sinensis* and seasons. It also takes many cleaning processes for dried *O. sinensis* to be applicable as a health tonic (30, 31). In addition, the harvested natural *O. sinensis* can be contaminated with relatively high levels of lead, arsenic, and copper (32). Because of this, several researchers have suggested the alternative use of biomass mycelial production as a substitute for natural *O. sinensis* (6, 33). Several fungal species contaminate the *O. sinensis* fruiting bodies (34). These cause unsuccessful biomass mycelium cultivation in general. Thus, the specific strain selection from natural *O. sinensis* was performed as the primary goal. Then, the mycelial biomass production and polysaccharide extraction were further applied. The bioactivities of this polysaccharide, such as anticancer, antioxidant, and immune cell activation, were evaluated in this study.

After isolation and molecular identification of fungal strains from 119 dried *O. sinensis* samples, only one specific strain of *O. sinensis* OS8 was obtained. In this finding, other strains were also found. Previous studies supported our results in which various fungal species contaminate the natural *O. sinensis* fruiting bodies (34). On SDA medium, *O. sinensis* OS8 was characterized in several aspects such as colony dense, downy, cottony, plumose, white to pale cream, with interwoven hyphae, dense aerial mycelium and hyphal strands, and margin plumose. Also, after microscopic observation, the branched hyphae, chaotic and interlaced, and rod-shaped spores, mostly 5 - 10 µm long, were recorded. These characteristics are similar to those of isolated *O. sinensis* strains (35). Since then, we considered and chose the *O. sinensis* OS8 as a well-known fungal medicinal mushroom for further investigation. Similarly, the success of specific strain isolation from natural *O. sinensis* was documented (15, 36). Our investigation further characterized new strains for mycelial biomass culture and their applications.

The large-scale production of mycelial biomass culture of this *O. sinensis* OS8 was performed. The submerged culture at 16°C in the dark light condition grew continually for 14 d. The medium composition is one of the main factors for mycelial biomass production. These different carbon sources such as glucose, yeast extract, and peptone, including nitrogen source, are suitable for mycelial growth and metabolite production on which many kinds of ascomycetes culture, including *Cordyceps* spp., are preferable (37, 38). In our submerged cultivation, the production yield and bioactive adenosine content from dried mycelial biomass were 23.61 g/l and 306.1 mg/100 g, respectively. The optimal growth was at a low temperature (16°C) and produced the highest mycelial biomass at more than 12 g/l (dried weight), as also found in mycelial cultivation of *O. sinensis (Hirsutella sinensis)*, which was isolated from natural *O. sinensis* (15). Although higher biomass was obtained in this study, some culture conditions, such as media and culture time, were not similar. Also in our case, a new strain of *O. sinensis* OS8 was used.

Nevertheless, adenosine as an essential bioactive compound was similarly detected when mycelia of *O. sinensis* was cultivated (15, 38). In our investigation, the bioactive chemical component of adenosine was detected in cultured *O. sinensis* OS8, which was remarkably higher than in natural *O. sinensis* (39, 40). Nucleosides are the major bioactive component in Cordyceps (41, 42). Nucleoside profiles, especially those of adenosine, have also been considered as chemical components for the quality control of cultured *O. sinensis* (39, 43).

Polysaccharides are the main component of *O. sinensis* (44). In our submerged culture, the polysaccharide yield extract from dried mycelial biomass of *O. sinensis* OS8 was 3.22 g/100 g. Several methods for polysaccharide extraction, including hot water extraction, Soxhlet extraction, ultrasonic-assisted extraction, and microwave-assisted extraction, are generally used (7). In this study, ultrasonic-assisted extraction was used; this obtained higher polysaccharides than other methods. A relatively low yield of extracted bioactive polysaccharide at about 486.16 mg/l was found from the mycelial culture of *C. sinensis* UM01 strain (45). Thus, the obtained polysaccharide yield might be different depending on several factors such as extraction methods or *O. sinensis* strains.

The bioactivities of polysaccharides are known to be tightly correlated to the structural feature of polysaccharides, upward to monosaccharide composition, α/β-configuration, glycosidic linkages, degrees of branching, and the length of side chains (46). Our present data showed that the novel fungus *O. sinensis* OS8 is enriched with β-D-glucan polysaccharides at a ratio of 56.92% from a total D-glucans, which contains 92.24%. It was similar to previous works which obtained β-D-glucan polysaccharides from cultured mycelial of *C. sinensis.* These were also correlated with the synergistic bioactive actions which exhibited antioxidant activity (9). Also, comb-like β-D-glucan can significantly induce immune organs and enhance the secretion of major cytokines such as TNF-α and IFN-γ (46). These obtained β-D-glucan polysaccharide configurations of cultured mycelial of a novel *O. sinensis* OS8 fungus were correlated to the bioactivities of anticancer, antioxidant, and immunomodulatory efficacies.

This study characterized chemical compounds from bioactive polysaccharides of OS8P using GC-MS. It is worth noting that the compounds of dodecamethyl pentasiloxane, 2,6-bis (methylthiomethyl) pyridine, 2-(4-pyrimidinyl)-1H-Benzimidazole, and 2-Chloro-4-(4-nitroanilino)-6-(O-toluidino)-1,3,5-triazine was first identified in the fermentation of mycelial *O. sinensis* OS8 as the main bioactive compounds. The chemical components between wild and cultured *O. sinensis* mycelia were reported. Among them are more abundant in mycelial cultivation, like myoinositol and some amino acids (6). In our study, these obtained chemical compounds from OS8P may correlate with its anticancer, antioxidant, and immune cell activation bioactivities.

The anticancer effect of OS8P on colon cancer cells by promoting cell apoptosis was investigated and the OS8P caused potent cell cytotoxicity to HT-29 cells. This cell cytotoxicity was clearly correlated with apoptosis, as evidenced in this investigation since the OS8P could induce DNA fragmentation in these colon cancer cells. Also, its morphological changes via AO/PI and DAPI-stained cells and SEM observation confirmed the colon cancer cell death through apoptosis. The SEM morphological changes of the colon cancer cells in our results were similar, in which the featured morphological variation of cell death apoptosis, such as condensation of chromatin, loss of microvilli, blebbing, and apoptotic bodies formation, was observed (25). It has been suggested that the OS8P might exert anticancer effects through modulating apoptosis. Apoptosis is a form of programmed cell death that results in the efficient removal of damaged cells. Most of the anticancer drugs currently used in clinical oncology exploit the intact apoptotic signaling pathways upward to trigger cancer cell death (47, 48). Until now, there has been no report about using polysaccharides from mycelial biomass culture of *O. sinensis* as a protectant against colon cancer. Indeed, few research groups have studied the anticancer effect of natural *C. sinensis* polysaccharide inhibiting colon cancer cell proliferation by apoptotic stimulation and autophagy flux blockage via mTOR signaling. The mechanism provides insight into the proliferative inhibition of colon cancer cells promoting useful polysaccharide applications as a therapeutic substance for colon cancer (49). In addition to this, the anticancer effect of the fractionated water-soluble polysaccharide obtained through *C. sinensis* mycelia on B16-F10 melanoma cells was also recorded (50). The results suggest that OS8P could be used as functional foods and/or as a new alternative bioprophylactic or biotherapeutic agent in colon cancer.

The development of cancer or human diseases is influenced by many factors, including free radicals (51). To protect against these factors, natural antioxidants obtained from fungi, especially from *O. sinensis* polysaccharides representing useful agents as functional foods and nutraceuticals, were valuably characterized. Antioxidant activities of the OS8P, the DPPH free radical scavenging activity, and the ABTS radical cation scavenging ability of the OS8P showed a positive and direct correlation with the sample concentration. Similarly, the exopolysaccharide produced from the mycelial liquid culture of the *C. sinensis* Cs-HK1 exhibited moderate antioxidant capacities with a Trolox equivalent antioxidant activity of 35 – 40 μmol Trolox/g (9). Nevertheless, the antioxidant activities of exopolysaccharide fractions isolated from the fermentation medium of the medicinal fungus *C. sinensis* showed a significant dependence on the protein content (52). The obtained antioxidant polysaccharides of OS8P might be applied for health protection and disease prevention.

The immunological response against human diseases, such as anticancer and infectious defenses, is comprised of cellular and humoral immunity, which is implicated or characterized by the influential functional role of T- and B-lymphocyte cells (13). The response of humoral immunity through B-lymphocyte was a specific reaction between antigens and antibodies. The cellular immunological defense has functioned specifically via T-lymphocytes (12). Splenocyte proliferation is the most direct indicator of immunoenhancement and is related to T- or B-lymphocyte immunity improvement. In fact, Con A induces T-lymphocyte cells, and LPS induces B-lymphocyte cell proliferation (53). This study obtained the effect of OS8P on splenocyte proliferation. The proliferation rate of the OS8P-treated cells was significantly higher than the control group and groups at 25– 200 µg/ml were significantly higher than the corresponding Con A or LPS control group. This confirms that the OS8P at a suitable concentration could significantly stimulate lymphocyte proliferation synergistically with Con A or LPS. This finding is consistent with previous reports showing that polysaccharides produced from *O. sinensis* have immunomodulatory responses (54). The potential of cultured *C. sinensis* polysaccharides as a prebiotic to reduce the side effects of cyclophosphamide on intestinal mucosal immunity was also postulated (55). This study’s induction of immune cell response suggests that OS8P might protect the human body against diseases, especially cancers.

## Data availability statement

The original contributions presented in the study are included in the article/supplementary material. Further inquiries can be directed to the corresponding author.

## Ethics statement

The animal study was reviewed and approved by the committee of permission of animal care and use for science and technology research, Maejo University.

## Author contributions

SC performed the investigation and wrote the original draft. MT was responsible for conceptualization, investigation, writing-review and editing, funding acquisition, and supervision. All authors contributed to the article and approved the submitted version.
